# Circadian oscillators in the epithalamus

**DOI:** 10.1016/j.neuroscience.2010.06.015

**Published:** 2010-09-15

**Authors:** C. Guilding, A.T.L. Hughes, H.D. Piggins

**Affiliations:** Faculty of Life Sciences, University of Manchester, Manchester, UK M13 9PT

**Keywords:** lateral habenula, medial habenula, ependymal, period 2, electrophysiology, bioluminescence, cpm, counts per minute, Hb, habenula, LD, light/dark cycle, LHb, lateral habenula, LHbL, lateral portion of the lateral habenula, LHbM, medial portion of the lateral habenula, LUC, luciferase, MHb, medial habenula, mRGCs, melanopsin-containing retinal ganglion cells, MUA, multiunit activity, NMDA, *N*-methyl-d-aspartic acid, PER2, period 2, PMT, photomultiplier tube, SCN, suprachiasmatic nuclei, TTX, tetrodotoxin, ZT, zeitgeber time

## Abstract

The habenula complex is implicated in a range of cognitive, emotional and reproductive behaviors, and recently this epithalamic structure was suggested to be a component of the brain's circadian system. Circadian timekeeping is driven in cells by the cyclical activity of core clock genes and proteins such as per2/PER2. There are currently no reports of rhythmic clock gene/protein expression in the habenula and therefore the question of whether this structure has an intrinsic molecular clock remains unresolved. Here, using videomicroscopy imaging and photon-counting of a PER2::luciferase (LUC) fusion protein together with multiunit electrophysiological recordings, we tested the endogenous circadian properties of the mouse habenula *in vitro*. We show that a circadian oscillator is localized primarily to the medial portion of the lateral habenula. Rhythms in PER2:: LUC bioluminescence here are visualized in single cells and oscillations continue in the presence of the sodium channel blocker, tetrodotoxin, indicating that individual cells have intrinsic timekeeping properties. Ependymal cells lining the dorsal third ventricle also express circadian oscillations of PER2. These findings establish that neurons and non-neuronal cells in the epithalamus express rhythms in cellular and molecular activities, indicating a role for circadian oscillators in the temporal regulation of habenula controlled processes and behavior.

The hypothalamic suprachiasmatic nuclei (SCN) are pivotal in controlling daily and circadian rhythms in physiology and behavior (Rusak and Zucker, 1979). The SCN circadian clock is synchronized to environmental light cues captured by rods, cones and melanopsin-containing retinal ganglion cells (mRGCs), and relayed directly to the SCN via the retinohypothalamic tract (Rollag et al., 2003; Guler et al., 2008; Hatori et al., 2008). Both *in vitro* and *in vivo*, rodent SCN neurons sustain circadian rhythms in spontaneous electrical activity, with peak firing rates recorded during the middle of the projected day (Brown and Piggins, 2007). Two key developments have led to a significant reappraisal of the extent of the mammalian brain's circadian system. First, studies of tissue from transgenic rodent models bearing bioluminescent (luciferase, or luc) reporters driven by clock genes/proteins, have unmasked a range of circadian oscillators of varying strength in other brain areas (Abe et al., 2002; Granados-Fuentes et al., 2004; Hiler et al., 2008; Guilding et al., 2009; Wang et al., 2009). Second, investigation of the central projections of mRGCs reveals that circadian photic information is directly conveyed to extra-SCN brain sites (Hattar et al., 2006). Collectively, these findings indicate that circadian processes in the brain are not exclusive to the SCN, and the identification of such extra-SCN sites is a key goal in circadian neurobiology (Guilding and Piggins, 2007).

One such candidate is the habenula (Hb). This epithalamic complex is anatomically divided into medial (MHb) and lateral (LHb) regions and it is implicated in learning, memory, attention, sleep/wake cycles and anxiety (Lecourtier and Kelly, 2007; Geisler and Trimble, 2008; Hikosaka et al., 2008). Building on earlier reports of retinal innervation of the rodent Hb (Cooper et al., 1993; Qu et al., 1996), mRGCs were recently found to innervate the mouse LHb region (Hattar et al., 2006). *In vivo* rat Hb neurons alter discharge activity in response to retinal illumination in a pattern resembling that of mRGC activation (Zhao and Rusak, 2005). Further, in *ex vivo* brain slices, LHb (but not MHb) neurons may sustain circadian rhythms in electrical activity (Zhao and Rusak, 2005). Thus, the rodent Hb has some SCN-like properties, but currently, there are no reports of rhythmic clock gene/protein expression in the Hb and therefore the potential for molecular circadian timekeeping properties in this structure remains unknown.

Here using videomicroscopy imaging and photon-counting of PER2::LUC fusion protein bioluminescence together with multiunit electrophysiological recordings, we investigate the endogenous circadian properties of the mouse Hb *in vitro*.

## Experimental procedures

### Animals

Adult male *mPer2*^*Luc*^ knock-in mice (PER2::LUC, University of Manchester breeding colony; Yoo et al., 2004) were maintained under a 12-h light/12-h dark (LD) cycle, with *ad libitum* access to food and water. Temperature was maintained at ∼18 °C and humidity at ∼40%. Zeitgeber time (ZT) 0 was defined as lights-on and ZT12 as lights-off. Animals were group housed for at least 2 weeks prior to experimentation. All procedures were carried out in accordance with the UK Animals (Scientific Procedures) Act 1986.

### Culture preparation

**Suppl. Fig. S1:**
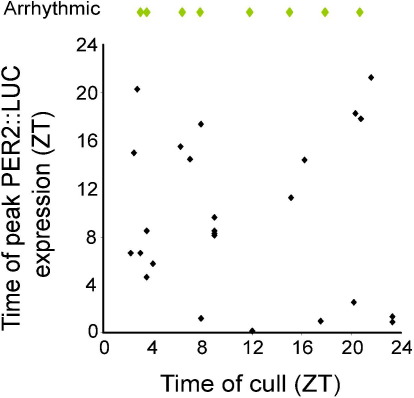


Mice were culled by cervical dislocation following halothane anesthesia (Concord Pharmaceuticals, Essex UK), at a range of times spanning the LD cycle (ZT 2.3–23.3 inclusive, Suppl. Fig. S1) to enable assessment of the effect of the time of culture preparation on the phase of peak PER2::LUC activity. For procedures conducted during the dark period, animal handling and brain extraction were conduced with the aid of night vision goggles to prevent exposure of animals to visible light. Coronal SCN or mid bilateral habenula (corresponding to the region between ∼−1.70∼−2.10 mm bregma; Paxinos and Franklin, 2001) slice cultures (300 μm thick) were prepared, and micro-dissected tissue was cultured as previously described (Hughes et al., 2008; Guilding et al., 2009).

### Luminometry

Total bioluminescence was recorded for up to 12 days from individual brain slice cultures with photomultiplier tube (PMT) assemblies (H8259/R7518P; Hamamatsu, Welwyn Garden City, UK) housed in a light-tight incubator (Galaxy R+, RS Biotech, Irvine, Scotland) maintained at 37 °C. Photon counts were integrated for 59 s every 1 min. All bioluminescence data were detrended by subtracting a 24 h running average from the raw data and smoothed with a 3 h running average.

### Bioluminescence imaging

Bioluminescence emission was imaged with an Olympus LV200 luminescence microscopy system (Olympus, Japan) fitted with a cooled Hamamatsu C9100-13 EM-CCD camera using a 20×0.4 NA Plan Apo objective (Olympus). The LV200 incubator was maintained at 37 °C in darkness. A transmitted light image was recorded prior to the start of each imaging run to aid anatomical localization of bioluminescence. Acquired images were transferred to ImageJ (version 1.37a, NIH, USA) and a region of interest tool was used to delineate discrete areas (MHb, LHb, ependymal cells of the third ventricle, and single cells) and assess relative bioluminescence over time. Putative single cells were identified and distinguished from background noise and isolated cosmic events by their characteristic size, shape and temporal expression profile.

### Tetrodotoxin and forskolin treatment

To assess the contribution of sodium-dependant action potential generation on the maintenance of bioluminescence rhythms in the Hb, explants were cultured with a voltage-gated sodium channel blocker, tetrodotoxin (TTX; 0.5 μM, Sigma, Poole, UK) in the medium. Tissue viability following damping of bioluminescence rhythms was assessed by treatment of cultures with the adenylate cyclase activator, forskolin (10 μM, Sigma), 3–8 days following culture. Treatment was performed as a complete medium change to fresh, forskolin-containing culture medium, otherwise identical to initial Dulbecco's Modified Eagle's medium (DMEM; Sigma) based culture medium.

### Extracellular recording

**Suppl. Fig. S2:**
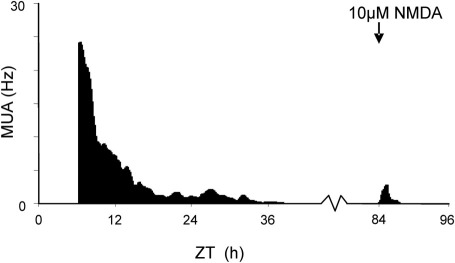


Habenula slice cultures (350 μm thick), corresponding to the same bregma location as used for the bioluminescence cultures, were prepared during the early lights-on phase (ZT 1–3) and maintained using methods similar to those described earlier (Brown et al., 2006). Slices were transferred to an interface style brain slice chamber continuously perfused (∼1.5 ml/min) with oxygenated (95% O_2_/5% CO_2_) aCSF supplemented with 0.0005% gentamicin (Sigma) and warmed to 36±1 °C. Slices were transilluminated and visualized under a dissecting microscope, and micromanipulators were used to precisely guide electrode tips onto the medial part of the LHb (LHbM; Fig. 5E). Extracellular multiunit activity (MUA) was recorded for at least 48 h, using aCSF-filled suction electrodes. Slice viability was tested 72–96 h after preparation by addition of a 5 min pulse of 10 μM (*N*-methyl-d-aspartic acid (NMDA; Sigma) to the perfusing aCSF; NMDA caused an acute elevation in cell discharge activity (Suppl. Fig. S2). Multiunit signals were differentially amplified (×20,000) and bandpass filtered (300–3000 Hz) via a Neurolog system (Digitimer, UK), digitized (25,000 Hz) using a micro 1401 mkII interface (Cambridge Electronic Design (CED), Cambridge, UK) and recorded on a PC running Spike2 version 6 software (CED).

Using Spike2, single unit activity was discriminated offline from these MUA recordings as previously described (Brown et al., 2006). Briefly, single units were discriminated on the basis of waveform shape, principal components-based clustering, and the presence of a clear refractory period in an interspike interval histogram. With these criteria we were able to successfully isolate up to two single units per recording.

### Data analysis

Molecular and electrophysiological rhythms were analyzed using curve fitting software (Clockwise, developed in house by Dr. T. Brown) as previously described (Bechtold et al., 2008). Processed bioluminescence data were assessed with Clockwise to determine the significance of circadian variation in PER2::LUC expression. Period (peak-peak and trough-trough averaged), phase (peak PER2::LUC expression during the interval between 24 and 48 h in culture), amplitude (peak-trough 24–48 h after culture) and rate of damping (the number of cycles observed before bioluminescence levels reached the level of dark current noise (±10%), previously determined for each individual PMT), were assessed manually by two experienced, independent researchers blinded to conditions. Period and phase measurements were subsequently confirmed with Clockwise and in all cases were found to be in close agreement with manually assessed data. Paired and unpaired *t*-tests (Excel; *P*<0.05 required for significance) were used as appropriate to determine statistically significant differences. Rayleigh analysis was used to assess clustering of the times of peak electrical and molecular activity (El Temps; Dr. A. Díez-Noguera, Barcelona, Spain, significance set at *P*<0.05).

## Results

We investigated circadian rhythmicity in the Hb using longitudinal electrophysiological recordings of neuronal activity and assessment of PER2::LUC bioluminescence emissions from adult mouse brain slice cultures *in vitro*. Both preparations enabled investigation of the endogenous circadian properties of the tissue since they are devoid of any input from the SCN or other known circadian oscillators.

### Circadian rhythms of PER2::LUC bioluminescence in the Hb complex

**Fig. 1:**
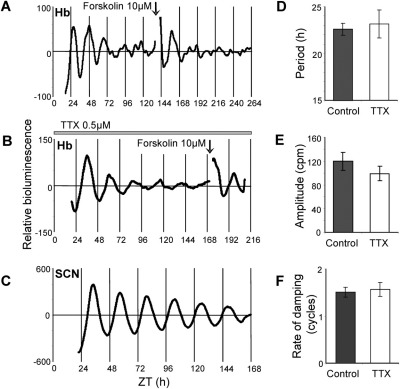
Circadian rhythms in PER2::LUC expression in Hb (A, B) and SCN (C) slice cultures. (A) Detrended PMT recording of PER2::LUC emission (counts per minute) in a Hb slice culture. Exposure to forskolin (10 μM) restarted oscillations. (B) Relative PER2::LUC bioluminescence in a Hb slice in the presence of 0.5 μM TTX. (C) PER2::LUC emission from an SCN slice culture. (E, F) Circadian characteristics of Hb slice cultures in control (*n*=34) and TTX containing medium (*n*=8), recorded in PMTs. There are no significant differences in period (D), amplitude (E) or rate of damping (F) following culture with 0.5 μM TTX.

**Fig. 2:**
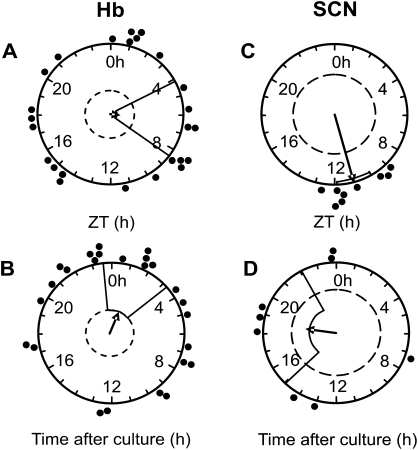
Rayleigh vector plots showing the phase of peak PER2::LUC expression *in vitro* recorded in PMTs, calculated as the time of peak bioluminescence after culture preparation or geographical ZT, in Hb and SCN slice cultures prepared at different times throughout the LD cycle. The phase of peak PER2::LUC expression in the Hb is not correlated with ZT (A) or time after culture preparation (B), while in the SCN it is correlated with ZT (C) but not time after culture preparation (D). Filled circles indicate the phase of peak bioluminescence in individual slice cultures. Direction of arrow indicates the mean phase vector, its length indicates the significance of phase clustering, with the surrounding box indicating the variance of phase. The inner broken line indicates the significance threshold of *P*=0.05.

To determine the circadian characteristics of the Hb complex, we performed long-term luminometry of PER2::LUC expression recorded in PMTs for up to 12 days. Seventy four percent of Hb slice cultures (25/34) displayed circadian rhythms in PER2::LUC emission, with a mean period of 22.65±0.6 h (Fig. 1; significance determined by Clockwise rhythm analysis software; *P*<0.05). Slices showed up to three circadian cycles in PER2::LUC bioluminescence before damping to apparent arrhythmicity (Fig. 1F; mean duration before damping 1.5±0.1 cycles). Forskolin, an activator of adenylate cyclase, is commonly used to evoke rhythms in damped circadian oscillators. Forskolin treatment (10 μM) restarted damped rhythms in all Hb slices monitored in PMTs (Fig. 1A; *n*=11). Rayleigh analysis of the phase of peak PER2::LUC in the Hb *in vitro* during the 24–48 h window after slice preparation revealed that peak phase was not significantly correlated with either ZT (Fig. 2A; *n*=25, *r*=0.105, *P*=0.759) or with time of culture preparation (Fig. 2B; *r*=0.333, *P*=0.064).

To assess the autonomy of PER2 rhythms in the Hb, we impaired action potential-dependent synaptic communication between cells with TTX. 0.5 μM TTX, a concentration which completely inhibits action potential production in the LHb (data not shown), did not alter PER2::LUC bioluminescence rhythms (Fig. 1B). Seventy five percent of Hb slices in TTX-containing media (6/8) displayed circadian rhythmicity, comparable to the percentage of rhythmic slices in non-TTX recording media (74%). The period of slices in TTX-containing media was 23.18±1.5 h, mean duration before damping was 1.6±0.2 cycles and the amplitude was 99±12.3 counts per minute (cpm). None of these circadian parameters were significantly different to those recorded from cultures maintained in normal medium (Fig. 1D–F; all *P*>0.05; unpaired *t*-test). The effects of forskolin stimulation persisted when the culture medium also contained 0.5 μM TTX, indicating that this action is also independent of sodium-dependent action potentials (Fig. 1B).

### Circadian rhythms of PER2::LUC bioluminescence in the SCN

**Suppl. Fig. S3:**
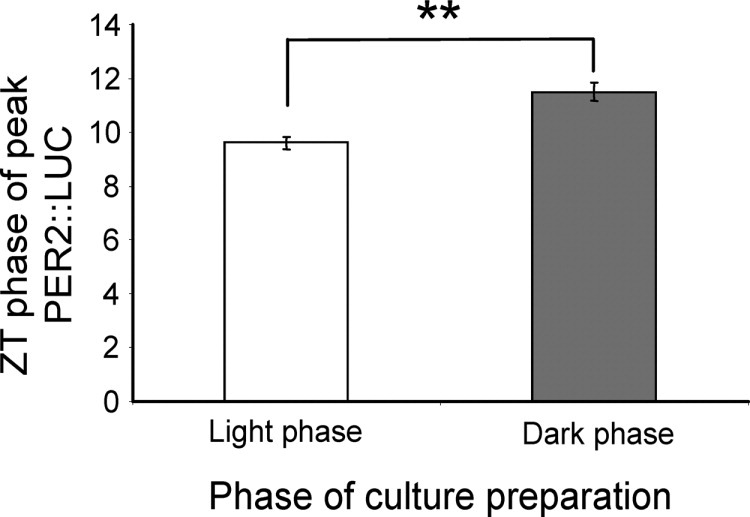


PMT recordings of PER2::LUC bioluminescence expression from SCN cultures (*n*=9) were all rhythmic, with peak bioluminescence at ZT10.9±0.4 and a mean period of 23.8±0.25 h (Fig. 1C). There was no significant difference in estimated period between SCN and Hb cultures recorded in PMTs (*P*>0.05) probably due to the variability of period between Hb slices, however, oscillations of PER2::LUC bioluminescence in the SCN were of significantly higher amplitude than in the Hb (SCN mean amplitude: 3995±660 cpm, Hb mean amplitude 119±15 cpm; *P*<0.00001) and were maintained for the full 7 days of recording, by which time oscillations had not damped to baseline (Fig. 1C). Rayleigh analysis revealed that peak phase of PER2::LUC expression was robustly correlated with ZT (Fig. 2C; *n*=9, *r*=0.948, *P*<0.00001) and not with time of culture preparation (Fig. 2D; *r*=0.0366, *P*=0.358). However, consistent with Yoshikawa et al. (2005), while peak phase did not correlate with a *specific* time after culture preparation, there was a significant effect of time of day of culture on the phase of PER2 expression: cultures prepared in the day consistently peaked earlier (Suppl. Fig. S3, peak phase 9.6±0.23 h) than those prepared at night (peak phase 11.5±0.33 h, *P*<0.01 versus day).

### Circadian rhythms of PER2::LUC bioluminescence visualized in the epithalamus

**Fig. 3:**
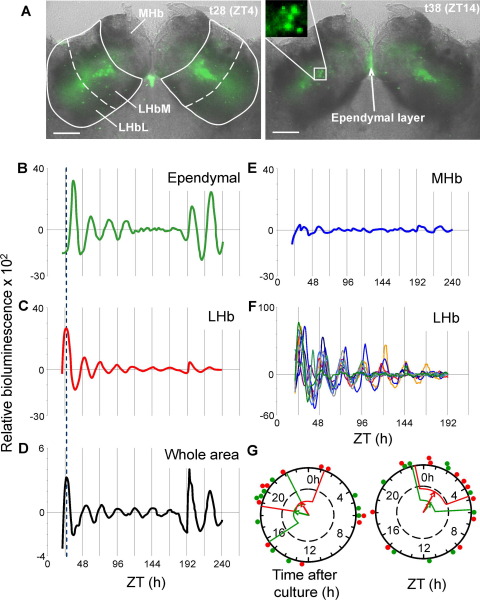
PER2::LUC expression in the habenula and ependymal cell layer. (A) EM-CCD images overlaid on a transmitted light image from a Hb slice culture, illustrating PER2::LUC bioluminescence (green) in the LHb and ependymal cell layer. Inset highlights single cells. Calibration bar 250 μm. Plots of relative PER2::LUC expression delineated in the (B) ependymal cell layer, (C) LHb, (E) MHb and, (D) integrated across the whole Hb culture. (F) Bioluminescence emission from representative individual cells in the LHbM. (G) Rayleigh vector plots showing the phase of peak PER2::LUC expression in vitro in the LHb and ependymal cell layer (green) calculated as the time of peak bioluminescence after culture preparation or ZT. Filled circles indicate the peak phase of individual slice (*n*=10).

To determine the anatomical location of PER2::LUC expression within the Hb complex, whole Hb slice cultures were imaged in real time with an EM-CCD camera. PER2::LUC expression was consistently visualized in the medial portion of the LHb (LHbM), in a central band radiating into the lateral portion of the LHb (LHbL) and in the ependymal cell layer lining the walls of the dorsal third ventricle (Fig. 3A, Movie 1). PER2::LUC bioluminescence was also observed in the MHb, adjacent to the dorsal third ventricle, though levels of expression here were much lower than in the LHb or ependymal cell layer.

**Fig. 4:**
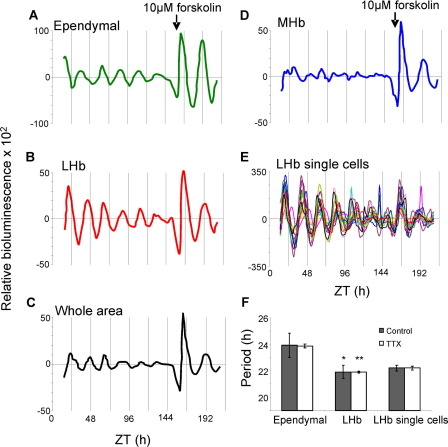
Circadian expression of PER2::LUC in the Hb complex persists in the presence of TTX (0.5 μM). Plots of relative PER2::LUC expression in slices cultured with TTX, delineated in the (A) ependymal cell layer, (B) LHb, (C) integrated across the whole Hb complex and (D) MHb. (E) Bioluminescence emission from individual cells in the LHbM. (F) Period of circadian oscillations in the ependymal cell layer, LHb and in single cells in the LHb in slices cultured in control (*n*=11) or TTX (*n*=3) medium. * *P*<0.05, ** *P*<0.01 versus the ependymal cell layer.

Continuous recordings of PER2::LUC activity were made from 11 slices for up to 10 days *in vitro*. Circadian oscillations of PER2::LUC bioluminescence were observed in the LHb in 10 cultures and in the ependymal layer in 8. (Fig. 3, Movie 1). The average period of oscillations differed significantly between the LHb and the ependymal cell layer (LHb: 21.3±0.5 h, ependymal: 23.9±0.9 h; *P*<0.05, *t*-test; Fig. 4F). This near 24 h periodicity in epithalamic ependymal cells is similar to that observed in mediobasal hypothalamic ependymal cells (Guilding et al., 2009). In the MHb, very weakly rhythmic temporal expression of PER2::LUC bioluminescence was observed in 5 of 11 cultures, and appeared linked to the waves of expression radiating up the ependymal cell layer (period 24±0.9 h; Movie 1, Figs. 3 and 4), and hence may reflect expression in ependymal tanycytes projecting into this structure (Cupedo and de Weerd, 1985). Forskolin treatment restarted damped rhythms in the ependymal cell layer to a much greater extent than in the LHb (Fig. 3), highlighting potential functional differences between these two oscillators.

Rayleigh vector plots of peak PER2::LUC bioluminescence indicated that the phase of the circadian rhythms did not correlate with ZT or the time after culture preparation in either the LHb (Fig. 1G; ZT: *r*=0.485, *P*=0.096, time after culture: *r*=0.281. *P*=0.457) or the ependymal cell layer (Fig. 1G; ZT: *r*=0.258, *P*=0.519, time after culture: *r*=0.269, *P*=0.491). While the small area of the ependymal layer generates clear circadian oscillations, the larger area of the LHb contributes most to the overall bioluminescence rhythm from the whole slice culture, demonstrated by the identical phasing of PER2::LUC rhythms in the LHb and in the whole tissue area (Fig. 3C, D). Single cells were visible in the LHbM in five of our recordings. Of 69 cells discriminated, 91% were rhythmic, with an average period of 22.2±0.2 h (Figs. 3F and 4F). The phases of peak PER2::LUC expression (measured at 24–48 h after culture) in single cells were significantly clustered in each individual slice (all *P*<0.05; Rayleigh analysis), which presumably underlies the whole tissue rhythmicity at this time. Very occasionally, faint cells were observed in the MHb; however these could not be tracked because luminescence here was at the edge of our detection limits (see Movie 1).

To examine the autonomy of molecular timekeeping in individual regions and cells from action potential dependent synaptic communication, we imaged Hb slice cultures in the presence of 0.5 μM TTX (*n*=3). Both the LHb and ependymal cell layer continued to display circadian rhythmicity following impairment of synaptic communication, with periods similar to those recorded in non-TTX treated slices (LHb: 21.9±0.07, ependymal: 23.9±0.15 h; *P*>0.05), yet still significantly different from each other (*P*<0.01, unpaired *t*-test, Fig. 4F). The weak oscillations in the MHb persisted in the presence of TTX, once again closely associated with the oscillations observed in the ependymal cell layer. Individual cells were visible in the LHbM in all of these recordings. Of 45 cells discriminated, 89% were rhythmic, with an average period of 22.4±0.14 h (Fig. 4F). These cells continued to express circadian rhythms for up to 9 days (the maximum duration of recordings), indicating that single cells are sustained oscillators which do not rely on sodium dependent synaptic communication for the maintenance of rhythms.

### Circadian variation in spontaneous discharge activity in the LHbM

**Fig. 5:**
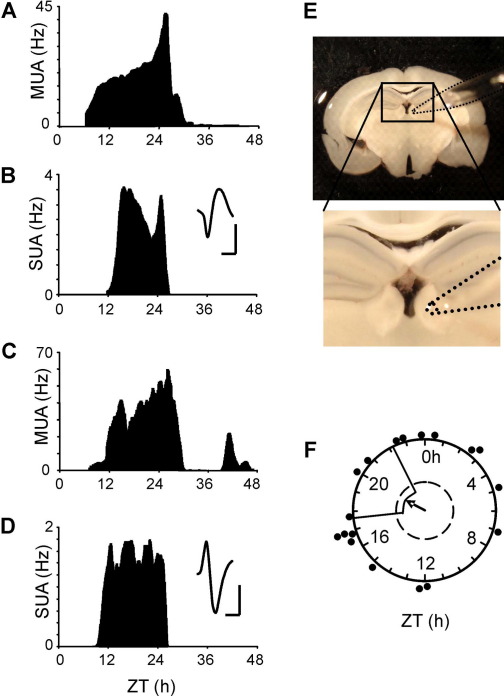
Temporal patterns of electrical activity in the LHb. Recordings from the LHb discriminated multiunit (MUA; A, C) and single unit electrical activity (SUA; B, D). MUA rhythms generally showed one peak (A) or peaks on consecutive days (C) with a circadian period. Single cells discriminated from the multiunit recordings in (A, C) are shown below them (B, D). Inset traces in (B, D) indicate the average spike waveforms for each cell; scale bars represent 15 μV (vertical) and 1 ms (horizontal). (E) Representative photograph of electrode positioning on the LHbM for electrophysiological recordings. (F) Rayleigh vector plot showing that the phases of peak electrical activity in the LHb are randomly distributed across the LD cycle. Filled circles indicate the phase of individual slices (*n*=18). For interpretation of the references to color in this figure legend, the reader is referred to the Web version of this article.

To determine if rhythmic expression of PER2::LUC in the Hb was accompanied by circadian variation in spontaneous discharge activity, population and single cell electrical activity were recorded extracellularly in the LHbM for at least 48 h *in vitro*. Consistent with previous studies in guinea-pig and rat LHb (Wilcox et al., 1988; Kim and Chang, 2005; Zhao and Rusak, 2005), we found neurons here to be spontaneously active. At least one distinct daily peak in firing rate activity was observed in 18/21 slices (Fig. 5A); the remaining three slices displayed clear spontaneous electrical activity, but no discernable individual daily peaks. Rayleigh analysis of the timing of multi- and single unit peak firing showed that there was no significant clustering of peak cellular discharge in relation to ZT (*n*=18, *r*=0.215, *P*=0.437; Fig. 5F), though more slices peaked during the night (*n*=12/21; Fig. 5) than during the day (*n*=6/21; Fig. 5).

Circadian oscillations were detected in multiunit discharge in five recordings (Fig. 5C; estimated mean period 24.0±2.0 h). Based on waveform shape, principal components-based clustering, and the presence of a clear refractory period in the interspike interval histogram, 22 single cells were discriminated offline from all recordings. These single cells fired spontaneously with a regular pattern of activity and tended to show only one peak in firing activity (Fig. 5B, D). This differs from SCN where cellular rhythms, although damping, can be monitored for up to 96 h *in vitro* (Brown et al., 2006).

## Discussion

Many cognitive and reproductive behaviors modulated by the Hb show circadian variations (Sutherland, 1982; Ralph et al., 2002; Hikosaka et al., 2008). Zhao and Rusak (2005) reported that rat LHb neurons show a circadian rhythm in spontaneous electrical activity when isolated from the SCN, but the potential molecular timekeeping properties of rodent Hb were unknown. Here we provide the first evidence of rhythmic clock gene expression in the Hb, localizing circadian oscillations in PER2::LUC bioluminescence to single cells in the LHb. We provide evidence of a functional output of these oscillations with the demonstration of circadian rhythms in neuronal excitability in a proportion of LHbM recordings. Further, we show circadian oscillations in PER2::LUC expression in the ependymal cell layer of the dorsal third ventricle. Circadian oscillations in the Hb complex do not appear to depend on action potential production since they persist in TTX-containing medium. Whole tissue rhythms, however, damp over time, as we and others have previously observed in other extra-SCN brain oscillators (Abe et al., 2002; Guilding et al., 2009; Wang et al., 2009), indicating that inputs from a master circadian oscillator and/or external cues are needed to maintain co-ordination of individual cellular rhythms and hence a coherent, high amplitude, tissue rhythm. The clear difference in the expression of PER2::LUC between the MHb and LHb further substantiates the view that these two regions of the Hb complex are anatomically and functionally very different in vertebrates (Amo et al., 2010; Zhao and Rusak, 2005; Quina et al., 2009).

Previous studies of rhythmicity in the Hb have utilized c-FOS as a marker of cellular activity. A daily rhythm of c-FOS immunoreactivity was observed in hamster and mouse LHbM, with significantly higher levels observed in the dark phase (Tavakoli-Nezhad and Schwartz, 2006). An earlier study in rat, however, found higher c-FOS in the light phase, indicating a possible species difference in circadian functioning in the LHbM (Chastrette et al., 1991). While one study has noted the presence of per1 and per2 mRNA in the rat MHb (Shieh, 2003), only one published study has investigated clock gene protein expression in the Hb, and did not detect either PER1 or PER2 protein expression in the hamster Hb (Tavakoli-Nezhad and Schwartz, 2006). We demonstrate for the first time that PER2 is rhythmically expressed in the LHb in mouse, and that this expression shows clear circadian variation and persists in isolation from the SCN.

Initial assessment of PER2::LUC rhythms in the Hb complex as a whole, revealed major differences in strength, robustness and phase compared to rhythms in the SCN; the SCN maintains high amplitude rhythms for the length of recordings, while rhythms in the Hb complex are of lower amplitude and damp rapidly. However, once individual regions were visualized and delineated, it became apparent that there are at least two different oscillators in the Hb complex, one localized to the LHb and one to the ependymal cell layer. Since these oscillators are randomly phased with respect to one another and have different periods, rhythm amplitudes observed in the Hb complex in PMTs, or when the whole area is delineated from images, are naturally reduced as compared to assessment of the individual areas separately (see Fig. 3). Interestingly, PER2::LUC rhythms in the LHb, ependymal cell layer and in single LHb cells were maintained after impairment of synaptic communication with TTX, indicating that individual cells can function as autonomous cellular oscillators. Continued cycling of individual cells in the presence of TTX, though with lower amplitudes, is a feature of SCN neurons (Yamaguchi et al., 2003) and of neurons in other recently described extra-SCN oscillators (Guilding et al., 2009).

Brain tissue differs in its phase-resetting properties after culture, related to the strength and characteristics of the endogenous circadian oscillators. In mice held under LD cycles, the SCN maintains a coherent phase locked to ZT when explanted *in vitro* (Fig. 2C). While this phase is not correlated to the specific time of culture (Fig. 2D), it is affected by it; slices prepared in the light phase peaked significantly earlier than those prepared in the dark phase (Suppl. Fig. S3). These data corroborate a comprehensive study by Yoshikawa and colleagues (2005), who demonstrated that the time of peak per1::luc expression in the SCN was delayed when cultures from rats previously held under a LD cycle were prepared in the night or early day versus the middle of the day.

In our study, the phase of oscillations in the Hb *in vitro* is not significantly correlated to ZT (Fig. 2A) or time after culture preparation (Fig. 2B), however, the correlation with time after culture preparation approached significance in PMTs (*P*=0.064), hence a larger sample size could conceivably reveal a significant effect. The phasing of the oscillations *in vitro* may result from complex interactions between the *in vivo* phase and resetting stimuli from the culture process. Extra-SCN tissues display a range of phase resetting responses following culture: explants of olfactory bulb from rat show random phase distribution of peak per1::luc bioluminescence, while other tissues from per1::luc rats are either unaffected or completely reset by culturing procedures (Abraham et al., 2005; Yoshikawa et al., 2005). Further, cultures from PER2::LUC mice containing the arcuate or dorsomedial nuclei of the hypothalamus are consistently reset by culturing procedures (Guilding et al., 2009).

A circadian rhythm in discontinuously sampled spontaneous neuronal discharge in the LHb both *in vivo* and *in vitro* was recorded in rat (Zhao and Rusak, 2005). In both settings, cells displayed peak firing rates during the projected day phase, similar to that observed in the SCN. We found circadian rhythms in population cell firing in 24% of our recordings from the LHbM *in vitro*, suggesting that in the mouse, the PER2 oscillator is weakly coupled to the electrical excitability of these neurons, although consistent with other tissues and cells, determining how rhythms in PER2::LUC relate to rhythms in physiological function remains to be determined. Unlike the study in rat, we did not see a consistent phase of peak cell firing in the day (Zhao and Rusak, 2005). Indeed a larger number of our slices peaked during the dark phase, corresponding with the phase of peak firing observed in many other extra-SCN brain regions (Inouye and Kawamura, 1979; Inouye, 1983). The discrepancies between these studies may result from the different preparation or sampling techniques used, or as seen for c-FOS rhythms, may be due to species differences in circadian physiology in the LHb (Chastrette et al., 1991; Tavakoli-Nezhad and Schwartz, 2006).

The transient and varied MUA rhythms detected in the mouse LHb *in vitro* contrasts with MUA recorded in the mouse and rat SCN where many neurons are capable of sustaining synchronized electrical rhythms *in vitro* for up to 96 h (Gribkoff et al., 1998; Albus et al., 2002; Brown et al., 2005, 2006). There are a number of possible explanations for this. One, the molecular oscillator in LHb neurons may only weakly target the membrane properties of these neurons such that many cells lack the unique intrinsic rhythm generation properties of SCN neurons (Brown and Piggins, 2007; Belle et al., 2009). Two, the anatomy of the LHb may isolate groups of cells and thus impede global synchronization of neuronal activity. The rat LHb has a complex anatomical organization (Geisler et al., 2003) and if organization of the mouse LHb approximates this then it is likely that there are distinct clusters of LHbM cells. In our investigations, we aimed our recording pipette in the LHbM, but it is probable that we monitored electrical activity from different LHbM cell populations in different slices and this may account for the heterogeneity in these MUA recordings. Three, the axons of LHb neurons project outside of the LHb and do not seem to have local collaterals (Kim and Chang, 2005), hence individual LHb cells may lack the coordinating activities present in SCN neuronal networks. At present it is not clear if one or all of these possibilities can account for the varied MUA in the LHbM and further experiments are required to resolve this.

The function of a circadian oscillator in the Hb is not known, although many behaviors which are modulated by the Hb such as sleep/wake cycles, stress responses, reproductive behaviors, pain responses, and reward-related learning show circadian variations (Chastrette et al., 1991; Corodimas et al., 1992; Haun et al., 1992; Landis et al., 1993; Nagao et al., 1993; Perissin et al., 2003; Matsumoto and Hikosaka, 2007; Webb et al., 2009). The Hb expresses receptors for neuropeptides associated with SCN efferents (Hernando et al., 2001; Cheng et al., 2006) as well as melatonin binding sites (Weaver et al., 1989), and retinal efferents arising from mRGCs innervate the LHb region (Hattar et al., 2006), indicating that photic and SCN-controlled output signals can regulate Hb cellular activity. Indeed, hamsters with split behavioral rhythms resulting from constant lighting conditions show asymmetrical c-FOS expression in the SCN and in the LHbM (Tavakoli-Nezhad and Schwartz, 2005). The Hb in turn projects to the pineal gland and raphe nuclei (Wang and Aghajanian, 1977; Herkenham and Nauta, 1979; Ronnekleiv and Moller, 1979; Araki et al., 1988), both of which are important for circadian timekeeping (Morin, 1999). Moreover, the raphe nuclei are implicated in major depression, an illness which can be precipitated or exacerbated by disruptions of circadian alignment through jet lag or shift work (American Psychiatric Association, 2000; Monteleone and Maj, 2008), and an expanding body of evidence highlights the functional importance of raphe-LHb pathways in major depression (Sartorius et al., 2010; Sartorius and Henn, 2007; Yang et al., 2008).

Of particular interest within our current data is the localization of individual cellular oscillators specifically to the LHbM. How the LHbM in mouse maps onto the Hb nuclei defined in rat (Andres et al., 1999; Geisler et al., 2003) remains to be determined. However, it appears to be this area, which expresses c-FOS in response to day-time but not night-time immobilization stress in rat (Chastrette et al., 1991), which exhibits a daily rhythm of c-FOS immunoreactivity in hamster and mouse (Tavakoli-Nezhad and Schwartz, 2006), and which expresses the asymmetrical c-FOS expression in response to splitting (Tavakoli-Nezhad and Schwartz, 2005). Further, this specific area receives the only major dopaminergic innervation into the Hb complex, which originates largely from the ventral tegmental area (VTA) (Herkenham and Nauta, 1977; Skagerberg et al., 1984; Gruber et al., 2007; Kowski et al., 2009). The LHb in turn projects to GABAergic neurons in the rostromedial tegmental nucleus (Jhou et al., 2009a). These neurons innervate VTA cells and negatively regulate dopaminergic neurons in the substantia nigra pars compacta (Jhou et al., 2009b), which are responsible for voluntary motor control and regulation of sleep patterns (Lima et al., 2007; Matsumoto and Hikosaka, 2007); suggesting a role for LHb-substantia nigra pathway in the circadian expression of voluntary locomotor activity and sleep/wake cycles. Indeed it is hypothesized that the LHb acts as a secondary oscillator regulating voluntary wheel-running in hamsters (Tavakoli-Nezhad and Schwartz, 2006).

## Conclusion

In summary, the Hb appears well placed to integrate photic and circadian information, and relay this downstream to influence circadian modulated behaviors. Our data suggest that the LHb can fine tune this circadian information with an endogenous clock.
